# Supplementary material for: A Critical View of the Application of the APEX Software (Aqueous Photochemistry of Environmentally-Occurring Xenobiotics) to Predict Photoreaction Kinetics in Surface Freshwaters
Source: Molecules. 2019 Dec 18;25(1):9. doi: 10.3390/molecules25010009 (PMC7017383; doi:10.3390/molecules25010009)
Supplement: Supplementary file 1 [file molecules-25-00009-s001.zip › 3.2.4_gcc-4.4.0.zip!/3.2.4_gcc-4.4.0/3.2.4_gcc-4.4.0/doc/octave/html/interpreter/Processing-Data-in-Cell-Arrays.html]

Processing Data in Cell Arrays - Untitled


Previous: Cell Arrays of Strings,
Up: Cell Arrays


---

#### 6.2.5 Processing Data in Cell Arrays

Data that is stored in a cell array can be processed in several ways
depending on the actual data. The simplest way to process that data
is to iterate through it using one or more `for` loops. The same
idea can be implemented more easily through the use of the `cellfun`
function that calls a user-specified function on all elements of a cell
array.

— Loadable Function: **cellfun** (name, c)  
— Loadable Function: **cellfun** ("size", c, k)  
— Loadable Function: **cellfun** ("isclass", c, class)  
— Loadable Function: **cellfun** (func, c)  
— Loadable Function: **cellfun** (func, c, d)  
— Loadable Function: [a, b] = **cellfun** (...)  
— Loadable Function: **cellfun** (..., 'ErrorHandler', errfunc)  
— Loadable Function: **cellfun** (..., 'UniformOutput', val)  
> Evaluate the function named name on the elements of the cell array
> c. Elements in c are passed on to the named function
> individually. The function name can be one of the functions
>
> `isempty`: Return 1 for empty elements. `islogical`: Return 1 for logical elements. `isreal`: Return 1 for real elements. `length`: Return a vector of the lengths of cell elements. `ndims`: Return the number of dimensions of each element. `prodofsize`: Return the product of dimensions of each element. `size`: Return the size along the k-th dimension. `isclass`: Return 1 for elements of class.
>
> Additionally, `cellfun` accepts an arbitrary function func
> in the form of an inline function, function handle, or the name of a
> function (in a character string). In the case of a character string
> argument, the function must accept a single argument named x, and
> it must return a string value. The function can take one or more arguments,
> with the inputs args given by c, d, etc. Equally the function
> can return one or more output arguments. For example
>
> ```
>           cellfun (@atan2, {1, 0}, {0, 1})
>           =>ans = [1.57080   0.00000]
> ```
>
> Note that the default output argument is an array of the same size as the
> input arguments.
>
> If the parameter 'UniformOutput' is set to true (the default), then the function
> must return a single element which will be concatenated into the
> return value. If 'UniformOutput' is false, the outputs are concatenated in
> a cell array. For example
>
> ```
>           cellfun ("tolower(x)", {"Foo", "Bar", "FooBar"},
>                    "UniformOutput",false)
>           => ans = {"foo", "bar", "foobar"}
> ```
>
> Given the parameter 'ErrorHandler', then errfunc defines a function to
> call in case func generates an error. The form of the function is
>
> ```
>           function [...] = errfunc (s, ...)
> ```
>
> where there is an additional input argument to errfunc relative to
> func, given by s. This is a structure with the elements
> 'identifier', 'message' and 'index', giving respectively the error
> identifier, the error message, and the index into the input arguments
> of the element that caused the error. For example
>
> ```
>           function y = foo (s, x), y = NaN; endfunction
>           cellfun (@factorial, {-1,2},'ErrorHandler',@foo)
>           => ans = [NaN 2]
> ```
>
>
>
>
> **See also:** isempty, islogical, isreal, length, ndims, numel, size.

An alternative is to convert the data to a different container, such as
a matrix or a data structure. Depending on the data this is possible
using the `cell2mat` and `cell2struct` functions.

— Function File: m = **cell2mat** (c)  
> Convert the cell array c into a matrix by concatenating all
> elements of c into a hyperrectangle. Elements of c must
> be numeric, logical or char, and `cat` must be able to
> concatenate them together.
>
>
>
> **See also:** mat2cell, num2cell.

— Built-in Function: **cell2struct** (cell, fields, dim)  
> Convert cell to a structure. The number of fields in fields
> must match the number of elements in cell along dimension dim,
> that is `numel (`fields`) == size (`cell`,` dim`)`.
>
> ```
>           A = cell2struct ({'Peter', 'Hannah', 'Robert';
>                              185, 170, 168},
>                            {'Name','Height'}, 1);
>           A(1)
>           => ans =
>                 {
>                   Height = 185
>                   Name   = Peter
>                 }
> ```
